# Concentric Needle Electromyography Findings in Patients with Ulnar Neuropathy at the Elbow

**DOI:** 10.3390/neurosci5040047

**Published:** 2024-12-10

**Authors:** Simon Podnar

**Affiliations:** Institute of Clinical Neurophysiology, Division of Neurology, University Medical Center Ljubljana, Zaloška cesta 7, 1525 Ljubljana, Slovenia; simon.podnar@kclj.si

**Keywords:** electrodiagnostics, needle electromyography, neuropathic changes, peripheral neuropathy, spontaneous denervation activity, ulnar neuropathy

## Abstract

In ulnar neuropathy at the elbow (UNE), the degree of neuropathic changes, the sensitivity of needle electromyography (EMG) in individual ulnar muscles, and the utility of individual EMG parameters are controversial. I compared qualitative needle EMG findings in two ulnar-innervated hands muscles and two ulnar-innervated forearm muscles in a group of previously reported UNE patients. Altogether, 170 UNE patients (175 arms) were studied. I found spontaneous denervation activity (SDA) most frequently in the first dorsal interosseus (FDI) (62%) and neuropathic changes in the abductor digiti minimi (ADM) muscle (88%). In the forearm muscles, SDA was more common (29% vs. 20%; *p* = 0.02), and neuropathic changes were similar in the flexor carpi ulnaris (FCU) and the flexor digitorum profundus (FDP) muscles. SDA and neuropathic changes were more common in the ulnar hand (88% and 77%) than in the ulnar forearm muscles (71% and 68%). Needle EMG is sensitive to diagnose UNE. For the detection of SDA FDI and neuropathic changes, ADM is the best muscle. Ulnar forearm muscles are less useful than ulnar hand muscles for UNE diagnosis.

## 1. Introduction

The most important test in the electrodiagnosis (EDx) of the ulnar neuropathy at the elbow (UNE) is the ulnar motor nerve conduction study (NCS). Motor NCS confirms UNE diagnosis and precisely localizes the lesion by demonstration of conduction block (>20%) or conduction velocity reduction [1]. The amplitude of the compound muscle action potential (CMAP) recorded from the abductor digiti minimi (ADM) or the first dorsal interosseus (FDI) muscle also indicates UNE severity. Information obtained from ulnar motor NCSs is complemented by ulnar sensory NCSs. Sensory nerve action potential (SNAP) recorded from the little finger provides objective information on the degree of the ulnar sensory fibers damage and helps to differentiate post-ganglionic from pre-ganglionic lesions of the primary sensory neuron (i.e., ulnar nerve lesion from C8 radicular lesion, respectively). In addition, needle electromyography (EMG) also has an important role in the evaluation of patients with UNE. It is particularly useful for the differentiation of UNE from lesions of the ulnar nerve fibers at other levels (e.g., C8 radiculopathy, ulnar neuropathy at the wrist, etc.). In addition, needle EMG of the ulnar hand and forearm muscles can provide information on the duration and severity of UNE (i.e., the extent of collateral reinnervation). Nevertheless, it is not clear which muscles need to be examined (i.e., sensitivity of needle EMG in individual ulnar muscles) [2,3,4,5] and which individual EMG parameters are most useful.

The present study aimed to compare the findings of qualitative needle EMG performed in individual ulnar muscles in a group of our previously reported UNE patients [6].

## 2. Materials and Methods

I performed a post-hoc analysis of the data obtained in a previously reported, prospectively recruited cohort of consecutive patients with clinical UNE diagnosis confirmed by EDx or ultrasonographic examination [6]. The inclusion and exclusion criteria for UNE patients were as previously described [6]. Patients with persistent typical UNE symptoms and abnormalities on clinical neurologic examination were included. The National Ethics Committee of Slovenia approved the study, and before the investigation, all participating patients provided written informed consent.

Demographic and clinical data were collected using the patient’s history and a focused questionnaire [7]. In all patients, clinical neurologic examination of both arms was performed. During needle EMG studies, the electromyographer was blinded to patients’ clinical information (i.e., history and findings of the clinical neurologic examination) but was aware of NCS findings.

NCSs have been performed using standard EMG equipment (Nicolet Synergy, Natus Medical Incorporated, San Carlos, CA, USA). Short segment NCSs (SSNCSs) were performed by stimulating the ulnar nerve at the wrist and in 2 cm steps from 4 cm distal (D4) to 6 cm proximal (P6) to the medial epicondyle (ME) of the elbow. Ulnar compound muscle action potentials (CMAPs) were recorded from ADM and FDI muscles. Concentric needle EMG of ADM, FDI, flexor digitorum profundus—ulnar part (FDP)—and flexor carpi ulnaris (FCU) muscles were performed. In each muscle, the abundance of spontaneous denervation activity (SDA) was semi-quantitatively described as 0—absent, 1—sparse, 2—moderate, and 3—dense. In addition, motor unit potentials (MUPs) were qualitatively assessed. During mild to moderate muscle activation, the amplitude (mV) of the highest MUPs was noted. MUP duration was described as 0—normal, 1+—mildly increased, 2+—moderately increased, and 3+—severely increased; polyphasicity was described as 0—normal, 1+—mild, 2+—moderate, 3+—pronounced. During stronger voluntary muscle activation, MUP recruitment and interference pattern (IP) density was graded as 0—normal, 1−—mildly reduced, 2−—moderately reduced, and 3−—severely reduced. During analyses, all SDA, MUP amplitude larger than 4 mV, MUP duration larger than 1+, polyphasicity larger than 1+, and recruitment reduction larger than 1− were regarded as abnormal.

I calculated parameter mean values (SDs). After checking parameter distributions, a non-paired two-tailed *t*-test was used for single comparisons of normally distributed data and the Mann–Whitney U-test was used for single comparisons of non-normally distributed data (MUP amplitude and polyphasicity). A z-test for two proportions was used for comparisons of proportions, and ANOVA was used for multiple comparisons. Statistical analyses were performed in a standard spreadsheet (Excel v 16.0, Microsoft, Redmond, WA, USA), and the Mann–Whitney U-test was performed using an online calculator [8] at a significance level of α = 0.05 (two-sided).

## 3. Results

**Figure 1:**
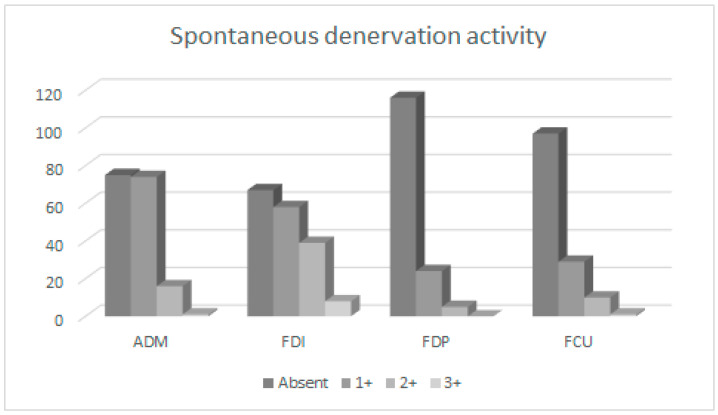
Comparison of frequency of spontaneous denervation activity (SDA) in four ulnar nerve-innervated muscles of 170 patients (175 arms) with ulnar neuropathy at the elbow (UNE). Note the higher frequency of SDA in both ulnar-innervated hand muscles compared to both ulnar-innervated forearm muscles. In each muscle, spontaneous denervation activity (SDA) was semi-quantitatively described as 0—absent, 1+—sparse, 2+—moderate, or 3+—dense. Note that in the majority of ulnar forearm muscles, no SDA was found. Legend: ADM—abductor digiti minimi; FDI—first dorsal interosseous; FDP—flexor digitorum profundus, ulnar part; FCU—flexor carpi ulnaris.

**Figure 2:**
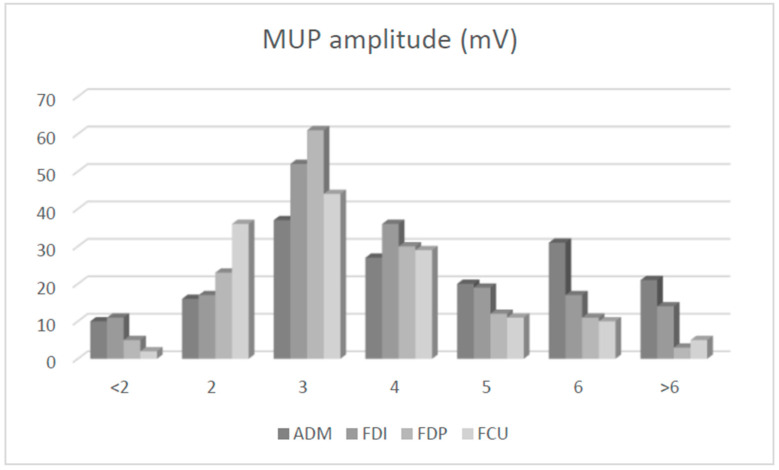
Comparison of motor unit potential (MUP) amplitudes in four ulnar nerve-innervated muscles of 170 patients (175 arms) with ulnar neuropathy at the elbow (UNE). In normal ulnar-innervated muscles, MUP amplitudes are usually 2–3 mV. Note the larger proportion of ulnar hand muscles in the pathologic range (i.e., MUP amplitude < 2 mV and >4 mV). Legend: ADM—abductor digiti minimi; FDI—first dorsal interosseous; FDP—flexor digitorum profundus, ulnar part; FCU—flexor carpi ulnaris.

Altogether, 170 UNE patients (175 arms) were studied. The patients’ mean age (SD) was 53 (15) years. The majority of included UNE patients (65%) were men, and the left arm was affected more often (66%) than the right (Table 1). CMAP amplitude on ulnar nerve stimulation 4 cm distal to ME and recording from ADM (mean (SD), 5.95 (3.75) mV) was similar to CMAP amplitude recorded from FDI (5.76 (4.23) mV; *p* = 0.36). On concentric needle EMG, SDA was more common and pronounced in both ulnar hand muscles (particularly in FDI) compared to both ulnar forearm muscles (*p* < 0.001; Table 2, Figure 1). The MUP amplitude was larger in both ulnar hand muscles (particularly in ADM) compared to both ulnar forearm muscles (*p* < 0.001; Figure 2). MUP duration was also highest in ADM (*p* < 0.001). By contrast, MUP polyphasicity was largest in the FDP muscle (*p* < 0.001) and similar in other ulnar-innervated muscles. Recruitment was most severely reduced in ADM muscle. Differences in needle EMG findings between individual ulnar muscles were significant (ANOVA, *p* < 0.001). Individual comparisons with lower significances (*p* > 0.01) are shown in Table 2.

The most sensitive needle EMG parameter was recruitment reduction (sensitivity: 56–83%), followed by SDA (sensitivity: 20–62%). Neuropathic MUP changes were more pronounced in both ulnar hand muscles compared to both ulnar forearm muscles. Likewise, the overall sensitivity of needle EMG in ulnar hand muscles was larger compared to ulnar forearm muscles (*p* < 0.01; Table 2). The highest sensitivity of qualitative needle EMG was found in ADM (88%), followed by FDI (77%). The cumulative sensitivity of needle EMG in our cohort was 96%.

The exact localization and underlying mechanism of UNE could be established in 167 arms. In 107 arms, UNE was due to external compression in the retrocondylar groove, and in 60 arms, it was due to entrapment under the humeroulnar aponeurosis (HUA); however, arms with UNE due to entrapment under the HUA were, as a rule, more severely affected in pairs of ADM vs. FDI and FDP vs. FCU muscle relations in the severity of neuropathic abnormalities were similar to each other and to the pooled cohort.

## 4. Discussion

The present study demonstrated a high sensitivity of qualitative needle EMG in a group of patients with a clinical diagnosis of UNE, confirmed by SSNCSs or ultrasonography. Overall sensitivities in individual ulnar-innervated muscles varied from 68% in FDP to 88% in ADM. Previous studies also demonstrated the high sensitivity of qualitative needle EMG in UNE, including evaluation of SDA, changes in MUP morphology, and MUP recruitment. In a study including 37 UNE patients, the sensitivity of needle EMG varied from 57% in FDP to 91% and 92% in ADM and FDI [4]. Another study including 441 ulnar neuropathies of different localizations and etiologies reported sensitivities of needle EMG in non-traumatic patients of 76% in FCU to 97% in ADM (FDP: 81%, FDI: 85%) [3]. The limitation of that study was the large variation in the frequency of needle EMG evaluations of individual ulnar muscles (from 4% for FDP to 59% for FDI) and a considerable proportion (29%) of ulnar neuropathies at other locations [3]. The current and previous studies thus demonstrated high sensitivities of qualitative needle EMG in UNE.

In the present study, I found SDA most often in FDI (62%), followed by ADM (53%) and ulnar forearm muscles (FCU: 29% and FDP: 20%; Figure 1). In a study of 25 UNE patients, SDA was found in 84% FDI, 52% ADM, 16% FDP, and 16% FCU muscles [2]. Another study reported somewhat lower and more uniform frequencies of SDA in different ulnar muscles: ADM, 43%; FDI, 30%; FCU, 24%; and FDP, 19% [4]. An even lower frequency of SDA (23%) was reported in a series of 116 arms with UNE confirmed by NCSs [9]. A probable explanation for such large differences might be the difference in the duration of UNE symptoms; in studies with lower proportions of arms with SDA, longer durations of symptoms would be expected. Unfortunately, information on the duration of UNE symptoms was not reported in previous studies. Another reason could also be the difference in UNE severity between studies. Nevertheless, SDA seems to be an important and common finding of needle EMG in subacute UNE.

All previous studies used SDA as the most reliable and unequivocal sign of ulnar nerve lesions. By contrast, authors of previous reports were divided on the utility of other qualitative needle EMG parameters. Stewart did not use “other criteria of “neurogenic” changes (size, configuration, and recruitment patterns of MUPs) because of the subjective nature of the interpretation of these findings” [2]. Another group agreed “that the interpretation of abnormal MUP configuration is more at risk for interobserver differences than the interpretation of SDA”, but believed that “for the diagnosis of UNE, abnormal MUP configurations are important” [9]. In the present study, I also used other qualitatively assessed needle EMG parameters. Nevertheless, to reduce the subjectivity of assessment, I raised the threshold for calling muscle abnormal to MUP amplitude > 4 mV and at least moderate and non-equivocal increase in MUP duration, polyphasicity (i.e., >1+ increase), and reduction in MUP recruitment (i.e., <1− reduction). In another study, MUP was considered chronic neurogenic if MUP peak-to-peak amplitude was ≥4 mV and/or MUP duration was ≥15 ms [10].

**Figure 3:**
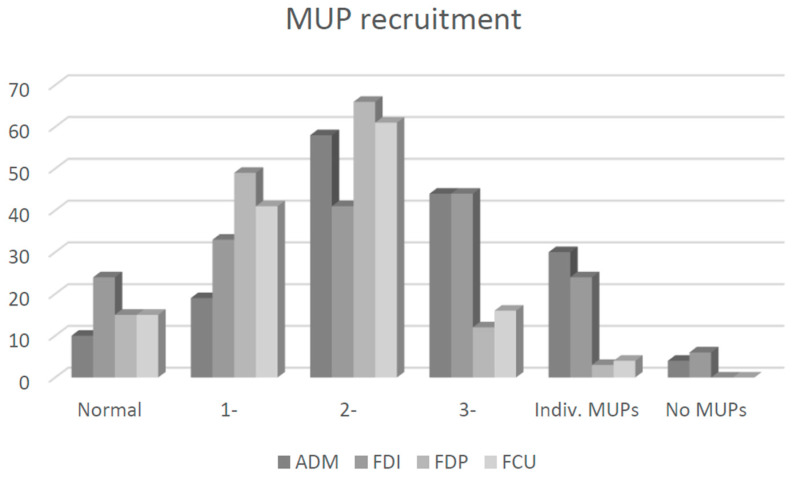
Comparison of motor unit potential (MUP) recruitment (i.e., interference pattern (IP) density) in four ulnar nerve-innervated muscles of 170 patients (175 arms) with ulnar neuropathy at the elbow (UNE). MUP recruitment was graded as 0—normal, 1−—mildly reduced, 2−—moderately reduced, 3−—severely reduced, individual MUPs, and no MUP recruitment. Note the larger proportion of ulnar hand muscles with severely abnormal MUP recruitment (i.e., MUP recruitment < 1−). Legend: ADM—abductor digiti minimi; FDI—first dorsal interosseous; FDP—flexor digitorum profundus, ulnar part; FCU—flexor carpi ulnaris.

The most sensitive needle EMG parameter in the present study was a reduction in MUP recruitment (56% to 83%; Figure 3), which is relatively straightforward to asses; i.e., high-frequency firing of a severely reduced number of MUPs is an unequivocal sign of neuropathic lesions. Therefore, a large majority of abnormal ulnar muscles in the present cohort would be identified by combining just two the most robust parameters: SDA and clearly reduced MUP recruitment. Other authors did not separately report this and other MUP parameters, and only a single group reported sensitivity of combined neuropathic MUP changes: FDI 70%, ADM 59%, FCU 51%, and FDP 31% [4].

In this study, the sensitivity of MUP amplitude and duration was moderate (18% to 46%), and the sensitivity of MUP polyphasicity was poor (5% to 20%). The lower utility of MUP amplitude and duration in the present study was probably due to a rather short time interval from the appearance of UNE symptoms to the EMG evaluation (usually only a few months). As a consequence, extensive collateral reinnervation could not have taken place yet in affected muscles. For unknown reasons, polyphasicity was much larger and more sensitive in FDP compared to other ulnar muscles (Table 2). This might be an intrinsic feature of this muscle.

Similar to previous reports, I found the sensitivity of needle EMG in both ulnar hand muscles to be higher compared to both ulnar forearm muscles, although the difference was not large (i.e., max: 20%; Table 2). One previous study reported an even smaller difference in needle EMG sensitivity of ulnar hand muscles (ADM 97%, FDI 85%) compared to ulnar forearm muscles (FDP 81% and FCU 76%) [3]; however, another study reported a larger difference: ulnar hand muscles 91% and 92%, and ulnar forearm muscles 57% and 65% [4]. As described earlier, Stewart also found SDA much more often in ulnar hand muscles (84% FDI, 52% ADM) compared to ulnar forearm muscles (FDP 16%, FCU 16%) [2]. This contrasts with another study that reported SDA in 43% FDI, 29% ADM, 29% FCU, and 20% FDP muscles [10]. In general, the present study confirms previous reports of a higher frequency of abnormal needle EMG findings in ulnar hand muscles compared to ulnar forearm muscles in UNE, although the difference is rather moderate. The exact cause of this difference is not clear. One possibility would be that the reinnervation of muscles closer to the lesion site is more efficient.

Based mainly on an influential previous report [2], it is generally considered that in UNE, FDI is more commonly and severely affected than ADM. Our results confirmed that SDA is indeed more common in FDI than ADM (Figure 1). However, our results also revealed more severe neuropathic changes in ADM compared to FDI (Table 2; Figure 2 and Figure 3). Similar relations were reported by others: sensitivity of needle EMG in ADM (97%) and FDI (85%) [3] and neurogenic changes in ADM (40%) and FDI (34%) [10]. Others did not report clear differences [9] or reported differences between both muscles that were minimal (sensitivity: ADM (53%) and FDI (49%)) [11]. Previous authors nevertheless recommend examining both FDI and ADM muscle because in 52% (SDA) to 67% (increased MUP polyphasicity) of arms with UNE, they found abnormalities in only one of these muscles [9]. The general conclusion is that SDA is more common in FDI (Figure 1), and neuropathic changes are more pronounced in ADM (Figure 2 and Figure 3). It is not clear what causes these differences. One possibility would be more efficient or earlier collateral reinnervation in ADM compared to FDI. This would lead to the earlier disappearance of SDA and more pronounced neuropathic changes in ADM. Previous attempts were also made to explain differences in ulnar muscle involvement in UNE based on differential involvement of fascicles within the ulnar nerve. It was suggested that the most frequently affected fascicles were those containing the nerve fibers for the small ulnar hand muscles, particularly the FDI, and from the terminal digital sensory branches [2]. At the elbow, these nerve fascicles lie deep in the ulnar nerve, adjacent to the bone, rendering them more susceptible to damage from external pressure [12]. It is not clear how such an arrangement affects the pattern of involvement in arms with ulnar nerve entrapment under the HUA; nevertheless, comparing the severity of neuropathic abnormalities between pairs of ADM vs. FDI and FDP vs. FCU between groups of arms with different underlying mechanisms of the lesion (i.e., external compression in the retrocondylar groove and entrapment under the HUA) did not show much difference in the present study.

Similarly, it is thought that in UNE, FCU is less often and less severely affected compared to other ulnar-innervated muscles. Again, this “FCU sparing” was thought to be due to the deep position of the fascicle-innervating FCU within the ulnar nerve [5]. Another explanation was that this “FCU sparing” was due to the separation of FCU branches from the ulnar nerve trunk proximal to the elbow. In previous studies including a few arms, FCU sparing was also found to correlate with the level of compression. Authors reported FCU abnormalities much more frequently in patients with retroepicondylar lesions than in those with entrapment under the HUA [5]. By contrast, in our patient series, FCU was more severely affected in arms with entrapment under the HUA, probably due to generally more severe lesions in this condition [13]. Anatomical studies also demonstrated that such proximal branching was rare, occurring in only 5% to 8% of arms [5]. Furthermore, even in these arms, branches destined to FCU accompanied the ulnar nerve trunk on its way through the retrocondylar groove and under the HUA [5]. The FCU was found to be normal or only mildly involved in 58% of arms with SDA in the FDI [5]. In the present study, FCU was actually more often and slightly more severely involved compared to the ulnar part of FDP, although differences were most of the time not significant (Table 2; Figure 1, Figure 2 and Figure 3). Others also found neurogenic changes slightly more often in FCU (29%) than in FDP (20%) [10]. As expected, patients with normal FCU had milder neuropathies than those with severely involved FCU [10]. Based on our report and several previous reports, we can conclude that in UNE, FCU is affected to a similar extent as the other ulnar forearm muscle—ulnar part of FDP.

Overall, the combined sensitivity of needle EMG analysis of four ulnar muscles (96%) in the present cohort was similar to the sensitivity of SSNCSs (97%) [6]. However, although UNE is usually diagnosed by finding a conduction block or conduction velocity reduction in a single elbow ulnar nerve segment, we do not usually diagnose UNE based on abnormal findings in a single ulnar innervated muscle.

The main limitation of the present study was the application of qualitative needle EMG parameters. This introduced a certain degree of subjectivity into the analysis. However, the effect of this was minimized by the application of rigorous criteria for muscle abnormality. Another important factor is that the same electromyographer performed all EMG examinations. This probably ensured a more consistent use of qualitative criteria. The limitation of the study was that before the needle EMG examination, the electromyographer performed SSNCSs and was aware of the findings. The strength of the study is the large number of included patients and arms with consistent inclusion and exclusion criteria. Also, the electromyographer was, at the time of needle EMG examination, not aware of the patients’ clinical information.

## 5. Conclusions

In arms with UNE, concentric needle EMG has a high sensitivity. For ulnar-innervated muscles, FDI has the highest frequency of SDA, and ADM shows (overall) the highest level of neuropathic abnormalities. I suggest performing needle EMG of FDI in subacute and EMG of ADM in chronic UNE. As a rule, ulnar forearm muscles are less severely affected than ulnar hand muscles, and FCU is affected to a similar extent as FDP. For qualitative EMG parameters, reduction in MUP recruitment followed by SDA is the most sensitive and probably also the most reliable.

## Figures and Tables

**Table 1:** Demographic features of patients with ulnar neuropathy at the elbow (UNE) included in the study.

Demographic Features	N (%)
Number of patients	170
Number of arms	175
Age, mean (SD)	53 (15)
Men, N (%)	110 (65)
Left arm affected, N (%)	115 (66)

N—number; SD—standard deviation.

**Table 2:** Comparison of qualitative concentric needle EMG parameters in ulnar muscles of patients with ulnar neuropathy at the elbow (UNE).

	A—ADM	B—FDI	C—FDP	D—FCU	*p*-Values > 0.05
Needle EMG sensitivity, P/N (%)	149/169 (88%)	134/175 (77%)	101/148 (68%)	99/140 (71%)	B/C = 0.09, B/D = 0.24, C/D = 0.65
Denervation activity, mean (SD)	0.66 (0.67)	0.94 (0.90)	0.23 (0.50)	0.38 (0.65)	-
Denervation activity, P/N (%)	93/169 (53%)	108/175 (62%)	30/148 (20%)	41/140 (29%)	A/B = 0.21, C/D = 0.08
MUP amplitude, mean (SD)	4.52 (2.25)	4.03 (2.10)	3.49 (1.30)	3.52 (1.57)	C/D = 0.43
MUP amplitude, P/N (%)	76/165 (46%)	55/169 (33%)	27/148 (18%)	26/139 (19%)	C/D = 0.92
MUP duration, mean (SD)	1.22 (0.88)	0.87 (0.88)	0.79 (0.67)	0.84 (0.81)	C/D = 0.28
MUP duration, P/N (%)	73/165 (44%)	50/169 (30%)	21/148 (14%)	32/139 (23%)	B/D = 0.19, C/D = 0.05
MUP polyphasicity, mean (SD)	0.24 (0.59)	0.22 (0.55)	0.62 (0.82)	0.23 (0.56)	A/B = 0.98, A/D = 0.77, B/D = 0.75
MUP polyphasicity, P/N (%)	12/165 (7%)	9/169 (5%)	30/148 (20%)	9/139 (6%)	A/B = 0.47, A/D = 0.79
Recruitment, mean (SD)	2.35 (1.53)	1.93 (1.92)	1.58 (0.86)	1.66 (0.92)	B/D = 0.22
Recruitment, P/N (%)	139/169 (83%)	118/175 (68%)	83/148 (56%)	83/139 (60%)	B/D = 0.17, C/D = 0.54

Non-significant comparisons of MUP parameters (Mann–Whitney U-test for MUP amplitude and polyphasicity and *t*-test for others) and their sensitivities (z-test) between ulnar muscles are shown (*p* > 0.05). P—abnormal muscles; N—number of all muscles; SD—standard deviation; CMAP –compound muscle action potential; MUP—motor unit potential; ADM—abductor digiti minimi; FDI—first dorsal interosseous; FDP—flexor digitorum profundus, ulnar part; FCU—flexor carpi ulnaris.

## Data Availability

The raw data supporting the conclusions of this article will be made available by the authors upon request.
